# Epidemiology of Viral Hepatitis and Liver Diseases in Nepal

**DOI:** 10.5005/jp-journals-10018-1128

**Published:** 2015-01-06

**Authors:** Ananta Shrestha

**Affiliations:** Department of Hepatology, The Liver Clinic, Liver Foundation Nepal, Tripureshwor, Kathmandu, Nepal

**Keywords:** Nepal, Hepatitis B virus, Hepatitis C virus, Hepatitis E virus, Epidemiology.

## Abstract

Geographical and ethnic diversity in Nepal has led to interesting epidemiology of viral hepatitis and liver diseases. Before the epidemic of Biratnagar that occurred in April to June 2014, Kathmandu was the only known endemic region for hepatitis E virus infection (HEV), where at least four previous epidemics have been documented since 1973. Due to rapid urbanization with still low socioeconomic condition, other major cities are appreciated to be at increasing risk of outbreaks. Characteristics of HEV epidemics and genetic changes of HEV during last decade have been studied. While hepatitis A virus infection was only common during childhood till recent years, it is re-emerging as an important etiology of acute hepatitis in young adults. Nepal is classified as low endemic region for hepatitis B virus infection (HBV) with overall seroprevalence of 1.1%. But, some ethnic groups and geographical areas have high prevalence rates. Despite low endemicity, HBV accounts for majority of cases of hepatocellular carcinoma and liver cirrhosis in the country. Similarly, though hepatitis C virus (HCV) is present in only 0.4% of general population, high seroprevalence was found among intravenous drug abusers many of them with HIV coinfection. Apart from hepatitis viruses, alcohol use also contributes significantly to liver cirrhosis and chronic liver failure. A unique form of hepatic venous outflow obstruction called ‘hepatic vena cava syndrome’ has been identified as an important etiology of liver cirrhosis and hepatocellular carcinoma.

**How to cite this article:**

Shrestha A. Epidemiology of Viral Hepatitis and Liver Diseases in Nepal. Euroasian J Hepato-Gastroenterol 2015;5(1):40-42.

## INTRODUCTION

Nepal is a small country sandwiched between India and China with population of 25 million people. Geographic and ethnic diversity within the country offers a unique epidemiology of liver disease in the region. Hepatitis E virus (HEV) infection is endemic in Kathmandu valley, with recurrent epidemics and sporadic cases in between. However, rural areas of Nepal appear to be spared. The epidemiology of acute hepatitis is changing over time. While hepatitis A virus (HAV) used to be very uncommon cause of acute hepatitis in adults till last decade; but, in recent years, it has been established as an important etiology in young adults. Prevalence of hepatitis B and hepatitis C virus (HBV and HCV) infection is low in Nepal. Marked heterogeneity is noted in prevalence of HBV among different ethnic groups and geographical regions.

## HEPATITIS E VIRUS INFECTION

Hepatitis E virus infection resulting in acute hepatitis is well studied and manifests in three epidemiological forms in Nepal: Epidemics, sporadic and outbreaks. It is mainly a disease of urban area and specifically Kathmandu valley. Very low prevalence of anti-HEV IgG is seen in the rural areas of Nepal compared to more than 90% of people above 50 years bearing the antibody in certain cities within Kathmandu valley.^1^ Five major epidemics have been documented so far during 1973,^2^ 1981 to 82,^3^ 1987^4^ 2005 to 2006 and 2014.^5^ First four being in Kathmandu valley and fifth one occurred out of Kathmandu valley in the city of Biratnagar in 2014. The characteristics of these epidemics are shown in Table 1. The epidemics of 1973, 1981 to 82 and 1987 occurred even before the discovery of HEV which was then labeled as NANB infection and later confirmed as hepatitis E on analysis of stored serum samples. It mainly affects young population 16 to 35 years and male predominance is noted. Attack rate of 1.4 to 4.6% of the population were observed during such epidemics with high mortality rate (25%) among pregnant women. In the last epidemic of Biratnagar, a clinical survey was done in Central Jail that hosted 656 inmates and 75 security personnel who shared the common water supply. Total 34 persons had symptoms of acute hepatitis leading to attack rate of 4.6%. Complications, like acute liver failure, subacute hepatic failure and cholestasis, are seen more frequently during epidemics. Prolonged cholestasis is seen more frequently in older age groups: 4.7% at 16 to 35 years *vs* 41°% in person aged >55. Overall, case fatality rate of HEV infection is 0.4%.

**Table 1:** **Table 1:** Characteristics of HEV epidemic in Nepal

*Year (place)*		*Estimated number* *of persons affected*		*Attack rate*		*Number of pregnant* *women reported*		*Mortality in pregnant* *women*		*Other observations*	
1973 (Kathmandu)		10,000		2.4%		118		25.4%			
1981-82 (Kathmandu)		12,000		1.4%		119		21%		27 nm VLP isolated in stool and transmitted in marmoset monkey, lasted 2 years	
1987 (Kathmandu)		7,405		—		73		24.6%		50% affected individuals were immigrants outside KTM	
2014 (Biratnagar)		8,500		4.6%		—		—		Nonimmune population to HEV. Sewage contamination of water supply. Shortlasting epidemic, lasted 2 months	

The epidemics occur in periodic fashion with interval in between when HEV manifested as sporadic cases. Hepatits E virus accounts for majority of cases during epidemics but, during sporadic forms, their proportion is only about 56%.^6^ Acute hepatitis without markers for hepatitis A to E is seen in nearly 34% during sporadic forms. Focal outbreaks occur in closed community like army training camps, prisons and boarding schools. These occur due to contamination of water source or the storage site.

Majority of HEV isolates from Nepal were genotyped as Ia except for some cases in 1996 to 1998 which were found to be of Ic. Minor genetic changes in HEV were also noted over time in isolates from 1997 to 2002.^7^ Five different clusters Ia-1 to Ia-5 were noted over this period. Ia-3 were the predominant isolates in 1997 which decreased over next few years and disappeared from 2000 onward. On the other hand, Ia-4 and Ia-5 emerged in 2000 and 2002 respectively.

## HEPATITIS A VIRUS INFECTION

Hepatitis A virus was known to be major etiology of acute hepatitis in children and foreign visitors to Kathmandu. Subclinical infections during childhood was so common that almost all children above 5 years showed anti-HAV IgG. Hepatitis A virus accounted for only 4% of adults with acute hepatitis in reports from 1986 to 2002 from Kathmandu.^4,6^ However in 2013, we noted that HAV was the major cause (40%) of acute hepatitis in adults and HEV accounted for only 13.3%.^8^ Improved food and water sanitation during childhood in recent years probably has rendered young population of Kathmandu vulnerable to symptomatic HAV infection. Further, past epidemics of HEV has provided herd immunity to the inhabitants of Kathmandu. Hepatitis A virus genotyping was done for the first time in isolates from 2013 and was found to be genotype IIIa.

## HEPATITIS B VIRUS INFECTION

Despite being landlocked by China and India with high and intermediate prevalence rate for hepatitis B infection respectively, Nepal remains to have low prevalence rate of 0.9%.^9^ Mode of transmission is predominantly horizontal during late childhood. Low infectivity state is noted in women of child bearing age and this has led to small carrier pool of hepatitis B virus. Prevalence of HBV infection is not uniform throughout the country. Marked variation is seen in different ethnic groups and geographical areas. Origin of the ethnic groups and socio-cultural practices prevalent in certain communities explains these heterogeneity in prevalence rates. While only 4% of cases of acute hepatitis is accounted by HBV, it appears to be the most important cause of HCC. Markers of HBV infection is seen in 46% of liver cirrhosis and 69% of HCC in Nepal.^10^ Eighty-five percent of chronic HBV infected individuals are HBeAg negative. Majority (56%) have HBV DNA < 2000 IU/ml but 27.8% of HBeAg negative individuals have HBV DNA > 2000 IU/ml [unpublished data]. Major genotype is D followed by genotype A which occured more frequently among patients with hepatocellular carcinoma (HCC).

### Liver Cirrhosis and Hepatocellular Carcinoma

Fifty-six percent of patients with chronic liver disease and HCC have HBV DNA positivity while only 48% have HBsAg positivity and rest accounting for occult HBV infection. Hepatitis C virus is seen in around 12% of patients with liver cirrhosis and HCC.^10^ A unique form of hepatic venous outflow obstruction—now known as hepatic vena cava disease—is prevalent in the country and, in combination with chronic HBV infection, is now accounted as the most important risk factors for development of HCC.^10^ Alcohol accounts for 23% of chronic liver disease when cut-off value of 80 gm/day of ethanol intake was considered significant.

Liver diseases in Nepal are yet to be explored, and the dynamics of changing epidemiology are important to appreciate.
